# Arthritis Caused by MRSA CC398 in Patient without Animal Contact, Japan

**DOI:** 10.3201/eid2612.202780

**Published:** 2020-12

**Authors:** Anders R. Larsen, Jesper Larsen

**Affiliations:** Statens Serum Institut, Copenhagen, Denmark

**Keywords:** livestock-associated methicillin-resistant *Staphylococcus aureus*, CC398, ST1232, Panton-Valentine leukocidin, immune evasion cluster, MRSA, CC398, clonal complex, bacteria, Japan, sequence type, staphylococci, antimicrobial resistance

**To the Editor:** In their recent article, Nakaminami et al. describe a case of human infection caused by Panton-Valentine leucocidin (PVL)–positive livestock-associated methicillin-resistant *Staphylococcus aureus* clonal complex 398 (MRSA CC398) in Japan (*1*). *S*. *aureus* CC398 includes 2 major MRSA variants with distinct genetic and epidemiologic properties, a highly transmissible and virulent human variant comprising both PVL-positive and PVL-negative strains and a more benign PVL-negative livestock-associated variant (*2*). We have previously shown that, in Denmark, nearly all case-patients colonized or infected with PVL-positive MRSA CC398 strains of the human variant have links to countries in mainland Asia, where the strain is endemic in the community (*3*). Our analysis revealed the existence of 2 phylogenetically distinct lineages (L1 and L2) with unique sequence types (STs), ST398 linked to China and ST1232 linked to Vietnam, Thailand, and Cambodia. Besides being PVL-positive and belonging to ST1232, the isolate described by Nakaminami et al. (*1*) also shared other genetic and phenotypic characteristics with the L2 strains: it carried *spa* type t034 and SCC*mec* type V and was resistant to aminoglycosides (gentamicin), lincosamides (clindamycin), macrolides (clarithromycin), and tetracyclines (tetracycline). We therefore suspect that the isolate belongs to the human variant of MRSA CC398.

In recent years, Denmark has witnessed increased importation of PVL-positive MRSA CC398 from mainland Asia because of international travel, in 1 case leading to a large hospital outbreak among mothers and infants in a maternity ward (*3*), and it seems possible that Japan and other countries might face a similar risk in the near future. Strain identification, source attribution, and knowledge about the transmission dynamics are essential for maintaining an effective MRSA infection control and prevention program. We therefore advocate using genotypic methods (e.g., as described by Stegger et al. [*4*]) that can accurately distinguish the human variant of MRSA CC398 from the livestock-associated variant.
